# Impact of a Multi-component Healthy Lifestyle Program on Job Performance Among Primary Healthcare Physicians in Tabuk City, Saudi Arabia: A Quasi-experimental Study

**DOI:** 10.7759/cureus.90046

**Published:** 2025-08-14

**Authors:** Wejdan Abdullah Alshehri, Mubarak Mohammed Alsaeed, Ahmad Raja Saeed Albalawi, Nadiyah Abdullah Almountashiri, Anwar Fahad Albalawi, Nagham Mohammad Alkhrissi, Wejdan Mohammed Alshehri, Arub Albalawi, Asma Ali Alharbi, Shouq Mawad Albalawi, Eman Agwa

**Affiliations:** 1 Family Medicine, Tabuk Health Cluster, Tabuk, SAU; 2 Public Health, Tabuk Health Cluster, Tabuk, SAU; 3 Training and Academic Affairs, Tabuk Health Cluster, Tabuk, SAU; 4 Public Health and Community Medicine, Benha University, Benha, EGY

**Keywords:** bmi, healthy lifestyle intervention, job performance, primary healthcare physicians, quasi-experimental study

## Abstract

Background: Primary healthcare physicians play a vital role in delivering frontline medical services, yet they are frequently exposed to high levels of occupational stress, poor lifestyle habits, and demanding workloads, which can negatively impact their job performance and overall well-being.

Objectives: This study aimed to assess the impact of a healthy lifestyle program on job performance.

Method: This quasi-experimental study was conducted on 170 primary healthcare physicians in the Tabuk region, who were selected by a multi-stage sampling technique, between January 1, 2025, and May 31, 2025. Data was collected using face-to-face interviews. Physicians were assessed using a structured questionnaire and job performance questionnaire, anthropometric measurements were obtained, and body mass index (BMI) was calculated.

Results: This study showed statistically significant improvement in dietary habits, physical activity, average sleeping hours, and job performance at the end of the intervention than before (p<0.001). BMI was significantly reduced after the intervention (p<0.001).

Conclusion: The implementation of a multi-component healthy lifestyle program resulted in statistically significant improvements in dietary habits, physical activity, sleep duration, and job performance. Additionally, a significant reduction in BMI was observed. So, these findings highlight the effectiveness of integrated lifestyle interventions in enhancing both the professional performance and overall well-being of healthcare providers.

## Introduction

The World Health Organization (WHO) defines health as more than just the absence of illness or disability; it also includes a condition of whole physical, mental, and social well-being. There are various forms of health, including financial, emotional, spiritual, mental, and physical. All of them enhance mental and physical well-being, reduce stress, and promote general health [1].

In order to efficiently organize and enhance national health systems and bring health and well-being services closer to communities, primary healthcare (PHC) is defined as a whole-of-society strategy that includes health promotion, disease prevention, treatment, rehabilitation, and more. PHC acts as the health system's gatekeeper [2].

The core members of the PHC workforce, doctors, are essential in coordinating an individual's needs for preventive, disease management, and curative treatment [3]. PHC providers will likely have to carry out the responsibility for providing healthcare services in order to achieve Universal Health Coverage (UHC) and develop the PHC system, especially during emergencies like the COVID-19 pandemic [4,5].

"A healthy employee is the cornerstone of a thriving business." This claim emphasizes how important workers are to the success of any company. Beyond just providing for fundamental needs, workplace health has a direct impact on job performance. Since most workers dedicate a large amount of their lives to their work, a safe and healthy workplace is essential to their ability to do their jobs well [6].

A number of research pertaining to suboptimal health-related behaviors have indicated a significant prevalence of physical and mental illness, job burnout [7], sleep difficulty, and even suicide among physicians [2].

Remaining physically active, eating a balanced diet, and quitting smoking could avoid almost 80% of chronic diseases and early deaths. Lifestyle decisions affect diabetes, cancer, stroke, dementia, and cardiovascular disease [8]. Compared to their healthy peers, unwell workers are more likely to be incapacitated, absent from work, and less productive. They also consume more healthcare resources [9].

There is growing evidence that physicians' personal health behaviors are significantly related to their professional effectiveness. Poor lifestyle choices have been linked to decreased cognitive function, lower energy levels, and worse decision-making, all of which can impact job performance [10,11]. These choices include physical inactivity, poor food, inadequate sleep, and unmanaged stress. On the other hand, adopting healthy lifestyle practices is linked to increased resilience, productivity at work, and mental and physical health [12,13].

Saudi Arabia is a rapidly developing country and faces a shortage of medical professionals [14]. Additionally, the majority of healthcare professionals experienced health issues that could have affected their work, and the majority of them made the decision to quit the company, which immediately raised the workload and stress on the remaining employees and led to poor quality of provided care [15]. A vital part in the population's health is played by healthcare professionals. Data on the effect of healthy lifestyle behaviors on healthcare workers' productivity in the Tabuk region is, however, lacking. Therefore, the goal of this study is to encourage PHC physicians to lead healthy lifestyles in order to enhance job performance in the Tabuk region. The objectives of this study were to (1) assess the baseline of lifestyle behaviors and job performance among PHC physicians prior to intervention, (2) implement a healthy lifestyle intervention program aimed at promoting physical activity, healthy eating, adequate sleep, and effective stress management strategies, and (3) evaluate the impact of the intervention program on changes in healthy lifestyle behaviors and job performance.

## Materials and methods

Study design and participants

This quasi-experimental study was conducted on 170 PHC physicians in the Tabuk region, Saudi Arabia, who were employed for at least six months prior to the study. Physicians who were pregnant or breastfeeding, had major health conditions that contraindicate participation in physical activity or dietary changes, had cognitive impairments, or had a history of substance abuse or dependence in the past six months were excluded. Data was collected during the period from January 1, 2025, to May 31, 2025. A multi-stage sampling technique was used, which involved dividing the Tabuk region into nine health sectors (King Fahd health sector, King Khalid health sector, Al-Wajh health sector, Duba health sector, Taima health sector, Abu-Rakah health sector, Umluj health sector, Al-Bidaa health sector, and Haql health sector). Of these, six health sectors were selected by a simple random sample. From each health sector, six PHC centers were selected by a simple random sample. The sample size was calculated using Epi Info version 7.2.5.0 (Centers for Disease Control and Prevention, Atlanta, Georgia, United States), assuming that the total number of doctors is 295 with a confidence level of 95%, an acceptable margin of error of 5%, and an expected frequency of 50%. The calculated sample size was 168 doctors.

One group of physicians involved were identified, and the items of the study were explained to them. An interview was arranged with those who agreed to participate in the study to complete the questionnaire and undergo a physical examination at baseline and five months following the intervention. Of 180 eligible physicians, 170 (94.4%) completed the study.

Data collection tools

A structured questionnaire composed of four sections, based on a face-to-face interview, was used to collect data. It included the following: (1) socio-demographic data such as age, gender, nationality, marital status, and academic degree, (2) lifestyle habits including questions about dietary habits, physical activity, smoking habits, and average sleeping hours, (3) questions about stress management skills, and (4) job performance using a self-reported individual work performance questionnaire (IWPQ), comprising five items related to task performance and eight items related to the contextual performance component, with each item scored on a five-point Likert scale from 1 (seldom) to 5 (always) [16-18].

For anthropometric measurements, weight and height were measured, and body mass index (BMI) was calculated according to standards [19,20]. The physicians participated in a multi-component program, that is, five months of intervention focusing on four key areas: (1) physical activity: weekly 60-minute group exercise and individualized physical activity plans developed with physiotherapists, (2) nutritional counseling: workshops on healthy eating and personalized dietary counseling, (3) sleep quality: educational sessions on sleep hygiene, sleep assessment, and consultation and encouragement of maintaining sleep diaries and regular sleep-wake cycles, and (4) stress management and mental well-being: stress management techniques, relaxation exercises, mindfulness training, cognitive behavioral therapy techniques, and individual stress counseling.

Adherence to the multi-component healthy lifestyle intervention was monitored and managed throughout the study period through direct supervision, attendance records, and weekly follow-up, and regular communication and reminders were used to assess and maintain participant engagement with the program components.

Delivery methods

Face-to-face interviews were conducted at the PHC centers. Also, online printed educational materials were delivered via a mobile app and emails.

Statistical analysis

The collected data were tabulated and analyzed using SPSS Statistics for Windows, Version 16.0 (SPSS Inc., Chicago, Illinois, United States). Categorical data were summarized as frequency and proportion, while quantitative data were presented as mean±SD. Appropriate statistical tests were used for comparison as the marginal homogeneity test, McNemar-Bowker test, Wilcoxon signed-rank test, and paired t-test. A p-value of ≤0.05 was considered statistically significant.

Ethical consideration

All participants provided informed written consent after clarifying the objectives, methods, benefits, and confidentiality of data. An approval from the Tabuk Institutional Review Board, Ministry of Health, Tabuk region, Kingdom of Saudi Arabia, was obtained to conduct this work (approval number: TU-077/024/281; date: December 31, 2024).

## Results

Table 1 shows that a total of 170 participants were included in the study. The majority were under the age of 30 (n=93; 54.7%). Males constituted 102 (60%), and 144 (84.7%) were Saudi. Regarding marital status, 93 (54.7%) were single. In terms of job title, the largest group were residents (n=56; 32.9%). Most physicians reported being non-smokers (n=149; 87.6%). The majority of physicians (n=129; 75.9%) reported knowledge of stress management skills. Among these, the most commonly recognized techniques were deep breathing exercises (n=99; 58.2%).

**Table 1:** Frequency distribution of primary healthcare physicians according to socio-demographic characteristics, smoking status, and stress management skills *More than one answer was allowed

Variables		Total number (170)	
		n	%
Age group (in years)	Under 30	93	54.7
	30-39	49	28.8
	40-49	23	13.5
	50-59	5	2.9
Gender	Male	102	60
	Female	68	40
Nationality	Saudi	144	84.7
	Non-Saudi	26	15.3
Marital status	Single	93	54.7
	Married	71	41.8
	Divorced	4	2.4
	Widow	2	1.2
Job title	General practitioner	48	28.2
	Resident	56	32.9
	Specialist	51	30
	Consultant	15	8.8
Smoking status	Smoker	12	7.1
	Former smoker	2	1.2
	Passive smoking	7	4.1
	Non-smoker	149	87.6
Knowing stress management skills	Yes	129	75.9
	No	41	24.1
Types of recognized stress management skills*	Deep breathing exercise	99	58.2
	Time management and planning	18	10.5
	Physical activity	67	39.4
	Positive self-talk	35	20.5
	Talking to a friend or a therapist	59	34.7
	Practicing mindfulness or meditation	28	16.4
	Progressive muscle relaxation	23	13.5
	Seeking medical help when needed	17	10
	Others	6	3.5

Table 2 shows that there were statistically significant improvements in dietary behaviors following the intervention as the proportion of participants reporting regular intake of a balanced diet, intake of multivitamin/food supplementation, dairy products, meat/fish, and vegetables/fruits weekly increased post-intervention, while those who skipped meals and had late night eating decreased post-intervention (p<0.001). Also, statistically significant improvements were observed in physical activity following the intervention. The proportion of physicians who previously engaged in physical activity only sometimes or rarely/never reported more frequent activity post-intervention (p<0.001).

**Table 2:** Comparison between nutritional status and physical activity before and after the intervention among primary healthcare physicians

Variables	Before the intervention	After the intervention				Marginal homogeneity test	P-value
		Regular	Often	Sometimes	Rarely/never		
		n (%)	n (%)	n (%)	n (%)		
Intake of balanced diet	Regular	15 (100)	0 (0)	0 (0)	0 (0)	84	<0.001
	Often	0 (0)	35 (74.5)	0 (0)	0 (0)		
	Sometimes	0 (0)	10 (21.3)	76 (75.2)	0 (0)		
	Rarely/never	0 (0)	2 (4.3)	25 (24.8)	7 (100)		
Skipping meals during the day	Regular	17 (100)	6 (7.3)	4 (8.5)	0 (0)	67	<0.001
	Often	0 (0)	76 (92.7)	9 (19.1)	0 (0)		
	Sometimes	0 (0)	0 (0)	34 (72.3)	0 (0)		
	Rarely/never	0 (0)	0 (0)	0 (0)	24 (100)		
Intake of multivitamins or food supplementation	Regular	35 (100)	0 (0)	0 (0)	0 (0)	21	<0.001
	Often	0 (0)	70 (83.3)	0 (0)	0 (0)		
	Sometimes	0 (0)	7 (8.3)	27 (100)	0 (0)		
	Rarely/never	0 (0)	7 (8.3)	0 (0)	24 (100)		
Intake of dairy products weekly	Regular	46 (79.3)	0 (0)	0 (0)	0 (0)	66	<0.001
	Often	12 (20.7)	63 (78.8)	0 (0)	0 (0)		
	Sometimes	0 (0)	13 (16.3)	29 (100)	0 (0)		
	Rarely/never	0 (0)	4 (50)	0 (0)	3 (100)		
Intake of meat/fish weekly	Regular	23 (100)	0 (0)	0 (0)	0 (0)	56	<0.001
	Often	0 (0)	59 (67.8)	0 (0)	0 (0)		
	Sometimes	0 (0)	25 (28.7)	49 (94.2)	0 (0)		
	Rarely/never	0 (0)	3 (3.4)	3 (5.8)	8 (100)		
Intake of vegetables/fruits weekly	Regular	52 (72.2)	0 (0)	0 (0)	0 (0)	78	<0.001
	Often	20 (27.8)	75 (89.3)	0 (0)	0 (0)		
	Sometimes	0 (0)	9 (10.7)	14 (100)	0 (0)		
Late-night eating	Regular	19 (100)	9 (16.7)	2 (3.8)	0 (0)	96	<0.001
	Often	0 (0)	45 (83.3)	16 (20.5)	0 (0)		
	Sometimes	0 (0)	0 (0)	59 (75.6)	0 (0)		
	Rarely/never	0 (0)	0 (0)	0 (0)	19 (100)		
Physical activity	Regular	16 (100)	0 (0)	0 (0)	0 (0)	37	<0.001
	Often	0 (0)	29 (65.9)	0 (0)	0 (0)		
	Sometimes	0 (0)	9 (20.5)	75 (85.2)	0 (0)		
	Rarely/never	0 (0)	6 (13.6)	13 (14.8)	22 (100)		

Table 3 illustrates that there was a statistically significant improvement in sleep duration following the intervention. Prior to the intervention, all participants slept less than eight hours per day; after the intervention, 26 (31.3%) of these individuals reported an increase to eight hours or more per day (p<0.001).

**Table 3:** Comparison between primary healthcare physicians regarding average sleeping hours before and after the intervention

Average sleeping hours per day	Before the intervention	After the intervention		McNemar-Bowker test	P-value
		Less than 8 hours	8 hours or more		
		n (%)	n (%)		
	Less than 8 hours	87 (100)	26 (31.3)	24.03	<0.001
	8 hours or more	0 (0)	57 (68.7)		

Table 4 demonstrates that statistically significant improvements were observed in task performance and contextual performance. The median task performance score increased from 20 before the intervention to 21 post-intervention. Similarly, the median contextual performance score rose from 29 to 31. There was a statistically significant reduction in BMI, with the mean value decreasing from 30.7±3.5 to 30.5±3.5 (p<0.001).

**Table 4:** Comparison of job performance and body mass index among primary healthcare physicians before and after the intervention *Paired t-test

Variables	Before the intervention	After the intervention	Wilcoxon signed-rank test	P-value
	Median (range)	Median (range)		
Task performance	20 (15-25)	21 (15-25)	5.9	<0.001
Contextual performance	29 (24-38)	31 (25-38)	8.1	<0.001
Body mass index	30.7±3.5	30.5±3.5	7.4*	<0.001

## Discussion

The impact of a multi-component healthy lifestyle intervention on different facets of PHC physicians' health behavior and work performance was evaluated in this study. Following the intervention, the results showed statistically significant increases in a number of areas, including sleep duration, physical activity, eating habits, and job performance.

Roughly 76% of participants reported they were aware of at least one stress-reduction strategy, and techniques including breathing exercises and physical activity were used more frequently. Since PHC physicians frequently experience chronic stress and burnout, stress management is essential to general welfare and productivity. Research indicates that stress-reduction behavioral regimens can effectively reduce burnout and improve job satisfaction. These findings are in line with previous studies carried out by Amoako et al. and Akintunde-Adeyi et al., showing stress management as one of the ways of improving the job performance of the employees in the organization [21,22].

This study demonstrated that dietary habits significantly improved as a result of the intervention. There was a statistically significant improvement (p<0.001) in the frequency of consuming a balanced diet, fruits and vegetables, dairy products, fish/meat, and fewer late-night meals. Significantly, post-intervention intake of regular multivitamins and dietary supplements rose. This is a factor that is known to boost immune function and reduce fatigue, both of which may support work performance. These findings are in accordance with Scapellato et al., who reported improved eating habits among healthcare workers with a higher intake of fruits, vegetables, and fiber, as also illustrated by Mecca et al. [23,24].

The present study showed that a statistically significant improvement in physical activity frequency was observed post-intervention (p<0.001), with a noticeable shift from sedentary behavior to frequent physical engagement. The importance of physical activity in enhancing job performance and workplace efficiency is well-documented [23-25]. These changes in lifestyle behaviors likely contributed to the enhanced job performance scores observed.

This study revealed that the average sleeping hours increased, with more physicians reporting ≥8 hours of sleep post-intervention (p<0.001). Sleep plays a fundamental role in physical health, cognitive functioning, and emotional well-being, all of which directly affect professional performance. This result is quite significant as physicians who lack sleep have been linked to decreased attention, memory problems, and a higher risk of medical errors. Improvements in this area certainly played a part in the changes in work performance [26].

This study revealed that there was a statistically significant reduction in BMI, which might be attributed to improved dietary habits and more engagement in physical activity. Other workplace studies targeting healthcare workers aiming at weight loss have shown significant weight loss [23,25]. Also, a non-randomized study by Rigsby et al., among 454 female employees at a hospital and nursing home, reported significant weight loss and a reduced mean body weight of 3.8 kg [27].

Considering job performance, the results of this study demonstrated a statistically significant improvement in both task and contextual job performance among PHC physicians following the implementation of a multi-component healthy lifestyle intervention. These improvements confirm the strong link between physician well-being and professional efficacy and highlight the value of investing in personal health to enhance workplace outcomes. Similar to this, Grimani et al. had a systematic review on the efficiency of workplace physical activity and nutrition programs in enhancing productivity, workability, and work performance; the review included 39 studies. Fourteen workplace nutrition and physical activity intervention studies yielded statistically significant changes on absenteeism, work performance, workability, and productivity [28].

Strengths and limitations

This study's primary strengths are as follows: its quasi-experimental study design, the multi-component intervention that targets multiple lifestyle behaviors rather than just one, the large sample size, the low dropout rate (5.6%), and the use of pre- and post-intervention assessments that enable within-subject comparisons.

This study has some limitations, namely, the absence of a control group, the use of self-report instruments to evaluate performance, which may constitute a bias, and short-term follow-up. In order to evaluate sustainability, future studies should incorporate objective performance measurements and a longer follow-up time.

Recommendations

Based on the significant enhancements reported in dietary habits, physical activity, sleep duration, and job performance, it is advised that similar wellness programs be incorporated into standard healthcare practice. Comprehensive, evidence-based interventions that target a variety of physician well-being factors, such as stress management, physical fitness, diet, and sleep hygiene, should be implemented by healthcare facilities. Organizational policies that foster work-life balance and a health-conscious culture should back these initiatives. Furthermore, maintaining good behavior changes and guaranteeing long-term effects can be achieved through the use of continuous monitoring and follow-up. Extending these programs to other healthcare environments and professional associations may boost employee output, lower burnout, and ultimately raise the standard of patient care. 

## Conclusions

The multi-component healthy lifestyle program significantly improved PHC physicians' sleep, physical activity, eating habits, and work performance. These results highlight how crucial it is to promote physician wellness initiatives, not only to safeguard healthcare physicians' health but also to enhance the overall quality of healthcare systems.
